# Lifestyle factors in patients with rheumatoid arthritis—a cross-sectional study on two Scandinavian cohorts

**DOI:** 10.1007/s10067-021-05905-2

**Published:** 2021-09-09

**Authors:** Julie Katrine Karstensen, Jette Primdahl, Maria L. E. Andersson, Jeanette Reffstrup Christensen, Ann Bremander

**Affiliations:** 1 Department of Regional Health Research, University of Southern Denmark, Odense, Denmark; 2 Danish Hospital for Rheumatic Diseases, University Hospital of Southern Denmark, Sønderborg, Denmark; 3 The DANBIO Registry, Copenhagen, Denmark; 4 Spenshult Research and Development Centre, Halmstad, Sweden; 5 Hospital of Southern Jutland, University Hospital of Southern Denmark, Aabenraa, Denmark; 6 Section of Rheumatology, Department of Clinical Sciences Lund, Lund University, Lund, Sweden; 7 Research Unit of General Practice, Department of Public Health, University of Southern Denmark, Odense, Denmark; 8 Research Unit of User Perspectives and Community-Based Interventions, Department of Public Health, University of Southern Denmark, Odense, Denmark

**Keywords:** Cardiovascular risk, Health behaviour, Lifestyle habits, Rheumatic diseases

## Abstract

**Introduction:**

The risk for cardiovascular diseases and other comorbidities increases with the number of unhealthy lifestyle factors in the general population. However, information on the combined number of unhealthy lifestyle factors in people with rheumatoid arthritis (RA) is scarce.

**Objectives:**

To study lifestyle factors and the association between disease impact and two or more unhealthy lifestyle factors in two Scandinavian cohorts with RA.

**Methods:**

We analysed data from two cohorts, Danish (*n* = 566; mean age 61.82 (SD 11.13) years; 72% women) and Swedish (*n* = 955; mean age 66.38 (SD 12.90) years; 73% women). Lifestyle factors (tobacco use, BMI, alcohol consumption and physical activity) were dichotomised as healthy vs. unhealthy (range 0–4 unhealthy factors). The association between disease impact and two or more unhealthy lifestyle factors was analysed using logistic regression.

**Results:**

Sixty-six percent of Danish and 47% of Swedish respondents reported two or more unhealthy lifestyle factors, most commonly, being overweight/obese and physical inactivity. For Danish participants, two or more unhealthy lifestyle factors were associated with (OR and 95% CI) male gender (1.86; 1.21–2.85), cardiovascular diseases (1.90; 1.28–2.82) and disease duration (0.97; 0.95–0.99). Corresponding findings for the Swedish cohort were male gender (1.42; 1.07–1.89), pain (1.10; 1.04–1.15), fatigue (1.09; 1.04–1.15), physical functioning (1.64; 1.28–2.10) and quality of life (0.35; 0.20–0.60).

**Conclusion:**

Many patients, most often male, in both cohorts had two or more unhealthy lifestyle factors. The number of unhealthy lifestyle factors indicates a multifaceted relationship with disease impact.

**Table:** 

**Key Points** *• This article contributes with new information concerning the proportion of patients with RA who have one or more unhealthy lifestyle factors.* *• Every second patient in the two included cohorts reported two or more unhealthy lifestyle factors.* *• Two or more unhealthy lifestyle factors were more common in men than in women but were not necessarily associated with measures of disease impact.* *• In order to adhere to a healthier lifestyle, a large proportion of the patients need to change more than one lifestyle habit, which entails a challenge for both the patients and the health professionals.*	

## Introduction

The prevalence of rheumatoid arthritis (RA) is similar in Denmark and Sweden, at circa 0.5–0.6% [1]. RA is a chronic, inflammatory and systemic disease that carries an increased risk of developing cardiovascular diseases (CVD) [2, 3]. The most pronounced symptoms in RA are pain, fatigue and physical disability, resulting in activity limitations and impaired quality of life [2]. Early and effective pharmacological treatment reduces the risk of damage to the joints and subsequent future disability and improves quality of life [4]. Pharmacological treatment targets the inflammation but lifestyle factors, such as smoking, being overweight, alcohol consumption and physical inactivity, may negatively affect inflammation or treatment [5–8].

Smoking enhances the risk of developing RA, exacerbates the disease progression [9] and negatively influences the treatment response [5]. Smoking is a well-known risk factor for CVD—a factor that is also applicable to people with RA [3]. Nearly 50% of the patients with RA are overweight or obese at disease onset, and an association between obesity and increased disease activity has been presented [6]. A high proportion of visceral fat can cause low degree inflammation, which increases the risk of CVD [6]. Knowledge about RA patients’ alcohol use and its effect on the disease is conflicting [7, 10]; however, alcohol may interact with some medications [11]. International studies have reported that only 14–29% of the patients with RA were physically active at a health-enhancing level [12, 13]. Physical inactivity has been associated with an adverse cardiovascular risk profile for people with RA [8]. The lack of adherence to a healthy lifestyle entails additional challenges when handling the increased risk for CVD found in patients with RA [14]. Two or more unhealthy lifestyle factors increase the risk for CVD and other comorbidities in the general population [15, 16]. The proportion of patients with RA who have more than one unhealthy lifestyle factor are also of great interest to health professionals working with lifestyle changes and cardiovascular screening.

International guidelines from the European Alliance of Associations for Rheumatology (EULAR) endorse a structured cardiovascular risk management for patients with RA and emphasise that all lifestyle recommendations for patients with RA should highlight the benefits of smoking cessation, regular physical activity and a healthy diet [3]. While evidence and recommendations support a healthy lifestyle for patients with RA to improve the effects of treatment and to lower CVD risks, most patients with RA prefer pharmacological treatment to lifestyle changes [17]. Habits are hard to change and the need to change multiple habits might seem overwhelming. Patients report that adhering to a healthy lifestyle is a ‘constant balance between ideality and reality’ that affects quality of life [18]. In patients with RA, the prevalence of smoking, alcohol consumption, dietary habits, obesity and physical activity are most often presented separately. Studies investigating the combined number of healthy vs. unhealthy lifestyle factors can be useful for understanding the challenges that people with RA may experience in lifestyle changes. Hence, the aim of this study was to investigate the combined number of unhealthy lifestyle factors. A secondary aim was to explore whether measures of disease impact were associated with two or more unhealthy lifestyle factors in people with RA. Our hypothesis was that a large proportion of patients with RA have two or more unhealthy lifestyle factors, and that measures of disease impact were associated with two or more unhealthy lifestyle factors.

## Methods

To obtain a wider perspective, we included RA cohorts from two Scandinavian countries — Denmark (*n* = 638) and Sweden (*n* = 1,061) — in this cross-sectional study. Information concerning four lifestyle factors — smoking, body mass index (BMI), alcohol consumption and physical activity — were available for both cohorts. Denmark and Sweden follow similar treatment plans concerning medication and lifestyle changes in patients with RA, based on the EULAR recommendations [3, 4, 19].

### The Danish cohort

The Danish cohort consisted of outpatients diagnosed with RA according to the American College of Rheumatology (ACR) criteria [20], who had participated in a cardiovascular screening consultation at the Danish Hospital for Rheumatic Diseases [19] between 2016 and 2018 (all patients with RA under the age of 75 are invited to undertake this consultation). All data were retrieved from DANBIO, a national Danish registry for patients with inflammatory diseases [21].

### The Swedish cohort

The Swedish data were retrieved from the BARFOT cohort (Better Anti-Rheumatic Pharmacotherapy) [22]. The BARFOT cohort includes patients with newly diagnosed RA, according to the ACR criteria [20], recruited between 1992 and 2006 from six rheumatology departments in Sweden for longitudinal follow-up studies (*n* = 2,837). In 2017, all living patients in the cohort were invited to participate in a survey concerning lifestyle habits and 68% (*n* = 1,061) completed the questionnaire [23].

### Lifestyle factors

Smoking habits were dichotomised by tobacco use or its absence. In the Swedish cohort, people who used snuff were registered as tobacco users since snuff is common in Sweden, but not in Denmark.

BMI was categorised as follows: underweight or a normal weight (< 25 kg/m^2^), overweight (≥ 25 and < 30 kg/m^2^) and obese (≥ 30 kg/m^2^). When categorised as healthy vs. unhealthy, we used the BMI limit of < 25 kg/m^2^ in contrast to ≥ 25 kg/m^2^.

Alcohol consumption was categorised as either below or above each national recommended limit. In the Danish cohort, alcohol was recorded as the number of units/week (max. 7 units/week for women and 14 for men) [19]. In the Swedish cohort, alcohol consumption was assessed using the Alcohol Use Disorders Identification Test—Consumption (AUDIT-C) questionnaire, yielding a summary score of 0–12 points [24]. According to the Swedish National Institute of Public Health, the limit for harmful drinking is AUDIT-C > 4 points for women and > 5 points for men (www.fhi.se).

Physical activity was gauged by reports of health-enhancing physical activity (of ≥ 150 min/week) or the lack thereof [25]. According to recommendations, physical activity of moderate intensity should be performed regularly for a minimum of 30 min for at least 5 days a week (150 min/week) or may be substituted with 20 min of vigorous activity for a minimum of 3 days per week (75 min/week) [25, 26]. In the Danish cohort, the number of days per week of moderate-intensity physical activity for at least 30 min was registered. In the Swedish cohort, physical activity (frequency and duration during the previous 7 days) was assessed via two categories: (1) moderate intensity (walking, gardening) and (2) vigorous activity (jogging or intense exercise) [27]. A combination of the two questions was calculated and then dichotomised into whether health-enhancing physical activity had been fulfilled or not.

### Measures of disease impact

The EurolQol-5 Dimensions (EQ-5D; 0–1, worst to best) provides a generic measure of health-related quality of life (HRQoL) [28]. The five-level version of EQ-5D was used in Denmark and the three-level version in Sweden. Physical functioning was measured with the Health Assessment Questionnaire (HAQ; 0–3, best to worst) [29]. Pain, fatigue and patient global assessment (PatGA) were measured using a visual analogue scale (VAS; 0–100, best to worst) in the Danish cohort and using a numeric rating scale (NRS; 0–10, best to worst) in the Swedish cohort [30]. The number of tender and swollen joints was based on the 28-joint count and was self-reported by the Swedish patients. In the Danish cohort, the 28-joint count stemmed from examination by a rheumatologist or a trained rheumatology nurse. In the Danish cohort, data on HAQ, HRQoL, pain, fatigue, PatGA and tender and swollen joints were not recorded during the screening consultations. To obtain this information, data were drawn from the most recent outpatient visit at the hospital, with a limit of 3 months before or after the screening consultation (consensus discussion).

Comorbidities were self-reported in both cohorts. The comorbidities were divided into two categories: CVD and pulmonary diseases. In the Danish cohort, hypertension, hypercholesterolemia, angina pectoris, myocardial infarction, stroke, vasoconstriction of the legs and ‘other CVDs’ were categorised as CVD and asthma, chronic bronchitis and tuberculosis were defined as pulmonary disease. In the Swedish cohort, hypertension, hypercholesterolemia, angina pectoris, myocardial infarction, thrombosis and stroke were categorised as CVD and asthma, chronic obstructive pulmonary disease and emphysema were considered pulmonary diseases.

### Statistical analysis

The combined number of unhealthy lifestyle factors were calculated in each cohort. Descriptive data were presented with mean (SD) or *n* (%), and the differences between the five groups (with 0, 1, 2, 3 and 4 unhealthy lifestyle factors) were analysed using one-way analysis of covariance (ANCOVA) and Fisher’s exact or chi-squared test for groups. ANCOVA post hoc analyses were performed with Bonferroni correction (*p* ≤ 0.005). To study the factors associated with two or more unhealthy lifestyle factors, the data were dichotomised into two groups (< 2 vs. ≥ 2 unhealthy lifestyle factors) and analysed by logistic regression analyses. The explanatory variables were HRQoL, pain, fatigue, PatGA, functioning and comorbidities. Age, gender and disease duration were included as possible confounders. Based on the minimal important difference for the EQ-5D of 0.05 [31], a sample size of 556 patients was needed to reach a power of 80% with an alpha of less than 0.05 (two-tailed). The statistical analyses of the Danish data were performed using STATA Version 15 (StataCorp, College Station, TX) and SPSS Version 22 (SPSS Inc., Chicago, IL) was used to analyse the Swedish data.

## Results

The final study cohort consisted of 566 Danish patients and, of these, 289 (46%) answered all questions on the same date as the screening consultation. Five patients had not answered all lifestyle questions and 67, for whom the most recent clinical visit fell outside the 3-month limit, were excluded. The mean age of the 72 excluded patients was 59 (SD 12.80) years and 69% were women.

The patients in the Danish cohort had a mean age of 61.82 (SD 11.13) years and a disease duration with a mean of 12.40 (SD 10.95) years and 72% were women. In the Danish cohort, the women were significantly younger and had longer disease duration and worse fatigue and physical functions than the men (Table 1). None of the participants in the Danish cohort reported having pulmonary diseases.

**Table 1:** General characteristics of the Danish cohort

			*N*	All *N* = 566	Men *N* = 159 (28%)	Women *N* = 407 (72%)
Age, years			566	61.82 (11.13)	64.20 (10.00)	60.90 (11.42)
Disease duration, years			561	12.40 (10.95)	10.61 (10.80)	13.04 (10.95)
Positive RF			340	249 (73.2)	65 (69.9)	184 (74.5)
DAS28-CRP			443	2.46 (1.10)	2.39 (1.05)	2.48 (1.10)
TJC			460	1.70 (3.61)	1.40 (3.30)	1.81 (3.73)
SJC			459	0.51 (1.52)	0.55 (1.60)	0.50 (1.50)
PatGa			547	34.13 (27.30)	31.72 (25.99)	35.08 (27.80)
Pain			542	31.33 (24.54)	29.62 (24.04)	32.01 (24.73)
Fatigue			547	38.91 (28.05)	34.20 (26.90)	40.80 (28.30)
HAQ			540	0.66 (0.62)	0.45 (0.52)	0.75 (0.64)
EQ5D			459	0.78 (0.17)	0.80 (0.17)	0.78 (0.16)
Cardiovascular disease^*1^			566	233 (41.2)	79 (49.7)	154 (37.8)
Pulmonary disease^*2^			0	0	0	0
Medical treatment						
	No DMARD or CS		532	0	0	0
	cDMARD ± CS		532	372 (70.0)	118 (76.6)	254 (67.2)
	bDMARD ± CS		532	155 (29.1)	35 (22.7)	120 (31.7)
	Only CS		532	5 (0.9)	1 (0.7)	4 (1.1)
Lifestyle factors						
	Tobacco use		566	125 (22.1)	39 (24.5)	86 (21.1)
	BMI, kg/m^2^		566	27.44 (5.70)	27.70 (4.80)	27.35 (6.00)
	BMI, kg/m^2^	< 25.0	566	209 (36.9)	49 (30.8)	160 (39.3)
	BMI, kg/m^2^	25.0–29.9	566	191 (33.7)	63 (39.6)	128 (30.4)
	BMI, kg/m^2^	≥ 30	566	166 (29.3)	47 (29.6)	119 (29.2)
	Alcohol above national limits^*3^		566	35 (6.2)	9 (5.6)	26 (6.4)
	Physical activity < 150 min/week (MVPA)		566	478 (84.4)	136 (85.5)	342 (84.0)

Data presented as mean (SD) or *n* (%)

*RF*, rheumatoid factor; *DAS28–CRP*, disease activity score in 28 joints–CRP and patient global assessment score (0–10); *TJC*, tender joint count (0–28); *SJC*, swollen joint count (0–28); *PatGa*, patient global assessment (0–100, best to worst); pain (0–100, best to worst); fatigue (0–100, best to worst); *HAQ*, Health Assessment Questionnaire (0–3, best to worst); *EQ5D*, Euroqol Five Dimensions (0–1, worst to best); *cDMARD*, conventional disease-modifying antirheumatic drugs (with or without corticosteroids); *bDMARD*, biological disease-modifying antirheumatic drugs (with or without corticosteroids); *CS*, corticosteroids; *BMI*, body mass index

^*1^Includes hypertension, hypercholesterolemia, angina pectoris, myocardial infarction, stroke, vasoconstriction of the legs and ‘other CVDs’

^*^^2^Includes asthma, chronic bronchitis and tuberculosis

^*3^Max. 7 units/week for women and 14 for men

In the Swedish cohort, 955 patients (90%) responded to all four lifestyle questions. The mean age of the excluded patients (*n* = 106) was 71.45 (SD 12.11) years and 69% were women. The final Swedish cohort had a mean age of 66.38 (SD 12.90) years and a disease duration with a mean of 15.55 (SD 3.85) years and 73% were women. As in the Danish cohort, women in the Swedish cohort were significantly younger and had worse fatigue and function than the men. Further, in the Swedish cohort, the women had a higher number of tender and swollen joints and worse pain and HRQoL than the men. There were also gender differences in CVDs, conventional disease-modifying antirheumatic drugs (DMARDs) and being overweight/obese, which were more common in men (Table 2).

**Table 2:** General characteristics of the Swedish cohort

			*N*	All *N* = 955	Men *N* = 259 (27.1%)	Women *N* = 696 (72.9%)
Age, years			955	66.38 (12.90)	69.39 (12.13)	65.27 (13.00)
Disease duration, years			955	15.55 (3.85)	15.72 (3.90)	15.48 (3.84)
RF pos			939	622 (65.1)	174 (67.2)	448 (64.4)
TJC			955	5.20 (6.60)	4.36 (6.20)	5.51 (6.70)
SJC			955	3.06 (4.90)	2.53 (4.72)	3.26 (4.94)
PatGa			955	3.04 (2.43)	2.82 (2.35)	3.12 (2.50)
Pain			923	3.42 (2.54)	2.94 (2.50)	3.60 (2.54)
Fatigue			927	4.08 (2.85)	3.40 (2.63)	4.33 (2.90)
HAQ			945	0.52 (0.55)	0.41 (0.53)	0.56 (0.56)
EQ5D			921	0.72 (0.25)	0.77 (0.21)	0.70 (0.25)
Cardiovascular disease^*1^			934	471 (49.3)	148 (57.1)	323 (46.4)
Pulmonary disease^*2^			919	113 (11.8)	35 (13.5)	78 (11.2)
Medical treatment						
	No DMARD or CS		884	146 (15.3)	35 (13.5)	111 (15.9)
	cDMARD ± CS		884	387 (40.5)	109 (42.1)	278 (39.9)
	bDMARD ± CS		884	293 (30.7)	66 (25.5)	227 (32.6)
	Only CS		884	58 (6.1)	23 (8.9)	35 (5.0)
Lifestyle factors						
	Tobacco use		955	163 (17.1)	63 (24.3)	100 (14.4)
	BMI, kg/m^2^		955	25.93 (4.73)	26.25 (3.62)	25.81 (5.10)
	BMI, kg/m^2^	< 25.0	955	444 (46.5)	99 (38.2)	345 (49.6)
	BMI, kg/m^2^	25.0–29.9	955	357 (37.4)	125 (48.3)	232 (33.3)
	BMI, kg/m^2^	≥ 30	955	154 (16.1)	35 (13.5)	119 (17.1)
	Alcohol above national limits^*3^		955	185 (19.4)	52 (20.1)	133 (19.1)
	Physical activity < 150 min/week (MVPA)		955	492 (51.5)	134 (51.7)	358 (51.4)

Data presented as mean (SD) or *n* (%)

*RF*, rheumatoid factor; *TJC*, tender joint count (0–28); *SJC*, swollen joint count (0–28); *PatGa*, patient global assessment (0–10, best to worst); pain (0–10, best to worst); fatigue (0–10, best to worst); *HAQ*, Health Assessment Questionnaire (0–3, best to worst); *EQ5D*, Euroqol Five Dimensions (0–1, worst to best); *cDMARD*, conventional disease-modifying antirheumatic drugs (with or without corticosteroids); *bDMARD*, biological disease-modifying antirheumatic drugs (with or without corticosteroids); *CS*, corticosteroids; *BMI*, body mass index

^*1^Includes hypertension, hypercholesterolemia, angina pectoris, myocardial infarction, thrombosis and stroke

^*2^Includes asthma, chronic obstructive pulmonary disease and emphysema

^*3^AUDIT-C > 4 points for women and > 5 points for men

### Unhealthy lifestyle factors

In the Danish cohort, 95% reported at least one unhealthy lifestyle factor and 66% reported two or more (Table 3). The corresponding numbers for the Swedish cohort were 82% and 47%, respectively (Table 4). In both cohorts, the most commonly reported unhealthy lifestyle factors were being overweight/obese and not adhering to physical activity recommendations. Sixty-three percent of the Danish patients and 54% of the Swedish ones were overweight/obese and 84% and 52% respectively, did not meet the recommendations for health-enhancing physical activity.

**Table 3:** Number of unhealthy lifestyle factors (0–4) in the Danish cohort (*N* = 566)

	0	1	2	3	4	*p*-value^*3^
	*N* = 30 (5.30%)	*N* = 163 (28.80%)	*N* = 294 (51.94%)	*N* = 72 (12.72%)	*N* = 7 (1.24%)	
Age, years	63.93 (8.86)	62.10 (12.05)	61.87 (11.23)	60.42 (9.37)	59.60 (10.60)	0.63
Gender, women/men	73.3/26.6	81.6/18.4	66/34	72.2/27.8	85.7/14.3	0.008
Disease duration, years	14.63 (12.01)	15.03 (13.53)	11.45 (9.32)	8.99 (8.53)	13.14 (11.51)	< 0.001
TJC	1.48 (4.20)	1.60 (3.30)	1.71 (3.60)	1.80 (4.23)	2.83 (4.20)	0.93
SJC	0.52 (1.83)	0.60 (1.50)	0.50 (1.63)	0.40 (1.01)	0.50 (0.84)	0.96
PatGA	31.87 (24.86)	33.64 (27.10)	34.23 (27.30)	35.60 (29.62)	36.71 (25.70)	0.97
Pain	30.73 (25.87)	31.70 (24.04)	30.92 (24.35)	32.30 (26.23)	33.14 (27.30)	0.99
Fatigue	33.70 (25.94)	38.20 (26.96)	38.80 (28.60)	42.90 (29.80)	42.90 (21.90)	0.61
HAQ	0.57 (0.52)	0.65 (0.62)	0.68 (0.63)	0.68 (0.63)	0.54 (0.51)	0.88
EQ5D	0.78 (0.15)	0.80 (0.18)	0.77 (0.16)	0.77 (0.17)	0.80 (0.15)	0.80
Cardiovascular disease^*1^	43.3	29.4	48	39	43	0.003
Pulmonary disease^*2^	0	0	0	0	0	na

Data presented as mean (SD) or %

*TJC*, tender joint count (0–28); *SJC*, swollen joint count (0–28); *PatGa*, patient global assessment (0–100, best to worst); pain (0–100, best to worst); fatigue (0–100, best to worst); *HAQ*, Health Assessment Questionnaire (0–3, best to worst); *EQ5D*, Euroqol Five Dimensions (0–1, worst to best)

^*1^Includes hypertension, hypercholesterolemia, angina pectoris, myocardial infarction, stroke, vasoconstriction of the legs and ‘other CVDs’

^*2^Includes chronic bronchitis, asthma and tuberculosis

^*3^ANCOVA or Fisher’s exact test

**Table 4:** Number of unhealthy lifestyle factors (0–4) in the Swedish cohort (*N* = 955)

	0	1	2	3	4	*p*-value^*3^
	*N* = 172 (18%)	*N* = 339 (35%)	*N* = 330 (35%)	*N* = 104 (11%)	*N* = 10 (1%)	
Age, years	63.97 (14.60)	66.59 (13.34)	68.29 (11.90)	63.25 (10.80)	70.70 (5.90)	0.000
Gender, women/men	77.3/22.7	75.5/24.5	72.7/27.3	59.6/40.4	50/50	0.005
Disease duration, years	15.78 (3.99)	15.52 (3.83)	15.57 (3.82)	15.34 (3.86)	13.80 (2.53)	0.547
TJC	4.65 (5.90)	5.21 (6.60)	5.36 (6.80)	5.54 (7.03)	5.30 (6.24)	0.795
SJC	2.47 (3.84)	2.88 (4.90)	3.50 (5.30)	3.42 (5.36)	1.50 (2.10)	0.125
PatGA	2.74 (2.32)	2.88 (2.40)	3.29 (2.50)	3.26 (2.50)	3.00 (1.83)	0.077
Pain	3.05 (2.50)	3.24 (2.44)	3.74 (2.63)	3.61 (2.64)	3.44 (2.01)	0.026
Fatigue	3.60 (2.90)	3.89 (2.82)	4.44 (2.90)	4.36 (2.74)	4.22 (2.90)	0.014
HAQ	0.36 (0.41)	0.50 (0.54)	0.60 (0.62)	0.57 (0.55)	0.54 (0.42)	0.000
EQ5D	0.77 (0.20)	0.73 (0.233)	0.68 (0.27)	0.69 (0.27)	0.76 (0.9)	0.001
Cardiovascular disease^*1^	36.6	47.1	59.7	53.4	70	0.000
Pulmonary disease^*2^	5.3	13.5	12.9	18.4	11.1	0.018

Data presented as mean (SD) or %

*TJC*, tender joint count (0–28); *SJC*, swollen joint count (0–28); *PatGa*, patient global assessment (0–10, best to worst); pain (0–10, best to worst); fatigue (0–10, best to worst); *HAQ*, Health Assessment Questionnaire (0–3, best to worst); *EQ5D*, Euroqol Five Dimensions (0–1, worst to best)

^*1^Includes hypertension, hypercholesterolemia, angina pectoris, myocardial infarction, thrombosis and stroke

^*2^Includes asthma, chronic obstructive pulmonary disease and emphysema

^*3^ANCOVA or Chi^2^

### Unhealthy lifestyle factors and disease impact

In the Danish cohort, significant differences were found between the five groups (of 0–4 unhealthy lifestyle factors) in gender, CVDs and disease duration. Two or three unhealthy lifestyle factors were more often reported by men than women and patients with two or three unhealthy lifestyle factors had a shorter disease duration than patients with only one unhealthy lifestyle factor (*p* = 0.008 and *p* = 0.001, respectively) (Table 3).

In the Swedish cohort, patients with two or more unhealthy lifestyle factors reported worse HRQoL (EQ5D-3L, *p* = 0.001) and worse physical function (*p* < 0.001) than patients with no unhealthy lifestyle factors. As in the Danish cohort, more men reported two or three unhealthy lifestyle factors than women and patients reporting three unhealthy lifestyle factors were younger than those reporting two unhealthy lifestyle factors (*p* = 0.005) (Table 4).

### Associations between disease impact and two or more unhealthy lifestyle factors

In both cohorts, men were at increased risk of having two or more unhealthy lifestyle factors (Denmark: OR 1.86, 95% CI 1.21–2.85; and Sweden: OR 1.42, 95% CI 1.07–1.89) in contrast to women. In the Danish cohort, two or more unhealthy lifestyle factors were associated with CVDs (OR 1.90, 95% CI 1.28–2.82) and disease duration (OR 0.97, 95% CI 0.95–0.99) (Table 5).

**Table 5:** Associations between the combined number of unhealthy lifestyle factors (UL) and disease-related factors among the Danish patients (*N* = 566)

	< 2 UL	≥ 2 UL	OR^†^ (CI 95%)	*P*-value
	*N* = 193	*N* = 373		
Age	62.35 (11.61)	61.54 (10.90)	0.99 (0.98–1.01)	0.75
Gender, men	38 (19.7)	121 (32.4)	1.86 (1.21–2.85)	< 0.001
Disease duration	15 (13.27)	11 (9.25)	0.97 (0.95–0.99)	< 0.001
TJC	1.60 (3.43)	1.74 (3.70)	1.01 (0.95–1.07)	0.80
SJC	0.57 (1.55)	0.50 (1.51)	0.95 (0.84–1.08)	0.45
PatGA	33.36 (26.70)	34.53 (27.65)	1.00 (0.99–1.01)	0.70
Pain	31.53 (24.30)	31.22 (24.71)	0.99 (0.99–1.01)	0.73
Fatigue	37.50 (26.80)	39.70 (28.70)	1.00 (0.99–1.01)	0.36
HAQ	0.64 (0.60)	0.67 (0.63)	1.33 (0.97–1.82)	0.07
EQ5D	0.80 (0.15)	0.78 (0.15)	0.80 (0.23–2.71)	0.71
Cardiovascular disease^*1^	61 (31.6)	172 (46.1)	1.90 (1.28–2.82)	0.001

Data presented as mean (SD) or *n* (%) if not noted otherwise

^†^Logistic regression, controlled for age, gender and disease duration. Data presented as odds ratio (OR) and 95% confidence intervals

*TJC*, tender joint count (0–28); *SJC*, swollen joint count (0–28); *PatGa*, patient global assessment (0–100, best to worst); pain (0–100, best to worst); fatigue (0–100, best to worst); *HAQ*, Health Assessment Questionnaire (0–3, best to worst); *EQ5D*, Euroqol Five Dimensions (0–1, worst to best)

^*1^Includes hypertension, hypercholesterolemia, angina pectoris, myocardial infarction, stroke, vasoconstriction of the legs and ‘other CVDs’

Additional findings in the Swedish cohort were the associations between two or more unhealthy lifestyle factors and CVDs (OR 1.83, 95% CI 1.40–2.43) and the number of swollen joints (OR 1.03, 95% CI 1.00–1.06). Two or more unhealthy lifestyle factors were also associated with global health (OR 1.08, 95% CI 1.03–1.14), pain (OR 1.10, 95% CI 1.04–1.15), fatigue (OR 1.09, 95% CI 1.04–1.15), function (OR 1.64, 95% CI 1.28–2.10) and HRQoL (OR 0.35, 95% CI 0.20–0.60) (Table 6).

**Table 6:** Association between the combined number of unhealthy lifestyle factors (UL) and disease-related factors among the Swedish patients (*N* = 955)

	< 2 UL	≥ 2 UL	OR^†^ (CI 95%)	*P*-value
	*N* = 511	*N* = 444		
Age	65.71 (13.8)	67.16 (11.71)	1.01 (0.99–1.02)	0.082
Gender, men	122 (23.9)	137 (30.9)	1.42 (1.07–1.89)	0.016
Disease duration	15.61 (3.88)	15.48 (3.81)	0.99 (0.06–1.02)	0.605
TJC	5.02 (6.36)	5.40 (6.79)	1.01 (0.99–1.03)	0.336
SJC	2.74 (4.56)	3.43 (5.23)	1.03 (1.00–1.06)	0.025
PatGA	2.83 (2.37)	3.27 (2.48)	1.08 (1.03–1.14)	0.004
Pain	3.17 (2.45)	3.71 (2.62)	1.10 (1.04–1.15)	0.001
Fatigue	3.79 (2.84)	4.41 (2.82)	1.09 (1.04–1.15)	< 0.001
HAQ	0.45 (0.51)	0.59 (0.60)	1.64 (1.28–2.10)	< 0.001
EQ5D	0.74 (0.22)	0.68 (0.27)	0.35 (0.20–0.60)	< 0.001
Cardiovascular disease^*1^	218 (42.7)	253 (57)	1.83 (1.40–2.43)	< 0.001
Pulmonary disease^*2^	53 (10.4)	60 (13.5)	1.34 (0.90–1.99)	0.151

Data presented as mean (SD) or *n* (%), if not noted otherwise

^†^Logistic regression, controlled for age, gender and disease duration. Data presented as odds ratio (OR) and 95% confidence intervals

*TJC*, tender joint count (0–28); *SJC*, swollen joint count (0–28); *PatGa*, patient global assessment (0–10, best to worst); pain (0–10, best to worst); fatigue (0–10, best to worst); *HAQ*, Health Assessment Questionnaire (0–3, best to worst); *EQ5D*, Euroqol Five Dimensions (0–1, worst to best)

^*1^Includes hypertension, hypercholesterolemia, angina pectoris, myocardial infarction, thrombus and stroke

^*2^Includes asthma, chronic obstructive pulmonary disease and emphysema

## Discussion

This study showed that a large proportion of patients with RA have two or more unhealthy lifestyle factors. This was more common in men but not necessarily associated with measures of disease impact. The most common unhealthy lifestyle factors were being overweight or obese and not meeting physical activity recommendations. In both cohorts, more than every second patient were overweight or obese and eight out of ten Danish patients and one in two Swedish patients did not adhere to the national recommendations for health-enhancing physical activity.

The findings in this study confirm the hypothesis as every second patient had two or more unhealthy lifestyle factors they would be recommended to be changed. In the general population, there is evidence that the risk for CVD and other comorbidities increases with the number of unhealthy lifestyle factors [15, 16]. The results from the present study and the already increased risk for CVD for patients with RA indicate that further research is needed on the association of a combined number of unhealthy lifestyle factors and risk for CVD in patients with RA.

Contrary to our hypothesis, two or more unhealthy lifestyle factors were not associated with measures of disease impact in the Danish cohort. Even though we found a significant association between several disease impact measures (swollen joints, patient global, pain, fatigue, function and HRQoL) and two or more unhealthy lifestyle factors in the Swedish cohort, only the difference in EQ-5D was clinically relevant, similar to findings in the general population [32]. Men had a higher risk of having two or more unhealthy lifestyle factors than women, which was supported by findings in both cohorts. This is also applicable to the general population in Denmark [33].

In the Danish cohort, the proportion of overweight patients (34%) was in line with the general Danish population (30%) [33]. However, obesity was almost twice as common in patients with RA than in the general population (30% vs. 17%, respectively) [33]. The numbers for the Swedish cohort accorded with the numbers from Swedish population data (36% overweight and 15% obese) [34]. Overall, a large proportion of the patients in both cohorts were overweight or obese. As there is a positive linear relationship between BMI and all-cause mortality, including diabetes, hypertension and CVD [35], a healthy BMI is of importance for patients with RA.

In patients with RA, there is evidence that moderate or vigorous physical activity, with adjustment to the person’s present aerobic fitness level, is beneficial and safe [25, 26]. General recommendations for health-enhancing physical activity from the American College of Sports Medicine and the American Heart Association are also applicable to people with rheumatic diseases [26]. In addition, the American Heart Association suggests that physical activity is just as important as smoking for an accurate cardiovascular risk score [25].

In the Danish general population, 29% do not meet recommendations for health-enhancing physical activity [33], which is why the finding in our cohort (85%) is alarming, considering the impact on general health and the risk for diabetes, hypertension and CVD. Correspondingly, 36% of the Swedish general population and 52% of the Swedish cohort did not meet the recommendations [34]. Overall, a large proportion of patients with RA do not meet the recommendations for health-enhancing physical activity. Management strategies for physical inactivity in patients with RA are of the utmost importance.

Smoking is the most prominent environmental risk factor for RA and the importance of smoking cessation should be emphasised [36]. In general, the proportion of smokers is higher in Denmark than in other Nordic countries [37]. In 2017, 22% of the Danish general population reported themselves as smokers [33], a proportion also found in our Danish RA cohort. In the Swedish general population, 18% reported tobacco use in 2020 [38], similar to our findings in the Swedish cohort (17%). Nevertheless, one out of five patients with RA still need to consider the cessation of tobacco use.

In the Danish cohort, only 6% reported harmful drinking [33] in contrast to 18% in the general Danish population. Treatment with the DMARD, methotrexate, requires alcohol restriction, which may explain this difference [11, 39]. However, in the Swedish cohort, 19% reported harmful drinking, which is in line with the general Swedish population (17%) [34]. An earlier study performed on the Swedish cohort found that patients who reported a harmful drinking pattern did not recall having had any discussions concerning alcohol with their healthcare professionals [23].

It is important to remember that lifestyle habits are modifiable risk factors. However, changing them is known to be a challenge for everyone, potentially affecting quality of life. The higher the number of unhealthy lifestyle habits, the bigger the challenge, according to people living with RA [40], who must also deal with the symptoms of living with a chronic inflammatory disease.

Guidance from healthcare professionals is of great importance to supporting behavioural change. EULAR recommendations encourage all health professionals to discuss lifestyle habits with their patients [3], but it is important to remember that the provision of information and advice alone does not lead to behavioural change [41]. In the Swedish BARFOT cohort, only half of the patients recalled having discussed physical activity with any health professional and one out of four patients recalled discussions about diet or smoking, while only one out of five recalled discussions concerning alcohol [23]. Supporting patients in behavioural change is also challenging for health professionals, who have expressed the need for additional education on the promotion of a healthier lifestyle in patients with RA [42].

### Strengths and limitations

The inclusion of two well-defined cohorts with systematically documented material is a strength of this study. We chose to include two Scandinavian RA cohorts to achieve a broader perspective on the combined number of unhealthy lifestyle factors among patients with RA. We compared lifestyles based on national recommendations in agreement with the EULAR guidelines [3]. There were some differences between the cohorts that should be addressed. The Swedish patients were slightly older, with a lower range in disease duration as a result of the inclusion period of 15 years in the BARFOT study; this might be one reason why disease duration was not associated with two or more unhealthy lifestyle factors, as found in the Danish cohort. The Danish cohort reflected patients from a specialist clinic who were invited to a cardiovascular screening consultation, to which only patients under the age of 75 are invited, whereas ages in the Swedish cohort ranged from 29 to 96.

The Danish Hospital for Rheumatic Diseases is the only centre in Denmark that systematically invites participants to cardiovascular screening consultation where lifestyle habits are simultaneously recorded which limited the sample size. The rather small sample size yielded a low number of patients classified as having none or all four of the selected unhealthy lifestyle factors — a condition that affected the ANCOVA in both cohorts, but especially the Danish cohort. We dichotomised the number of unhealthy lifestyle factors in the regression analysis, which did not change the findings for the two cohorts. We also performed a logistic regression analysis omitting the middle group (0–1 vs. 3–4 unhealthy lifestyle factors) as the dependent factor with similar results.

The sample size might also affect the generalizability of the Danish results. However, it is well known that a larger proportion of patients with RA are overweight/obese and less physically active than the general population [6, 12, 19, 43], supporting our findings. Self-reported data and use of specific limits and the cross-sectional design may not present the full picture, which is why all lifestyle habits should be discussed in a clinical meeting before guiding patients in behavioural change.

In this study, we used BMI to classify people as either healthy or unhealthy, in agreement with most population studies on lifestyle factors. However, people with RA have an increased risk of cachexia, which can also occur early in the disease process [44, 45]; this means that the use of the BMI categories recommended by national standards might misclassify some of the participants [46]. Patients with RA may have central obesity and a low fat-free mass index as a result of cachexia, corresponding to a lower BMI than 25 kg/m^2^, which is why body composition measures are recommended for a more accurate classification of the terms overweight and obesity [46].

Cardio-vascular diseases were more common in the group of patients with two or more unhealthy lifestyle factors as might be expected, Unfortunately we lack more detailed information concerning comorbidity duration and additional possible comorbidities.

## Conclusion

In addition to the burden of living with long-standing disease, a large proportion of the patients in our two RA cohorts reported more than one unhealthy, but modifiable lifestyle factor of importance for the risk for CVD. Every second patient with RA had two or more unhealthy lifestyle factors in both the Danish and Swedish cohorts—of which being overweight or obese and not meeting the recommendations for health-enhancing physical activity were the most common. Having two or more unhealthy lifestyle factors was more common in men than in women but was not necessarily associated with measures of disease impact. Our study supports that health professionals in rheumatology need to support healthy lifestyle changes in patients with RA and with a special focus on men and physical activity and dietary interventions.

## Code availability (software application or custom code)

Not applicable.

### Ethics approval (consent to participate consent for publication)

The Danish cohort was registered with the Danish Data Protection Agency (No.: 09200415). As this study is a registry-based study, patient consent was not needed according to Danish law. The local regional ethical committee was asked whether a formal approval was required and they confirmed by mail that the study did not need to be registered (No.: 20202000–79). All patients in the Swedish cohort provided informed consent and the lifestyle questionnaire was approved by the Swedish regional ethics committees (No.: DNR LU 2016/816).

## Data Availability

Not applicable.

## References

[1] Kiadaliri AA, Kristensen LE, Englund M (2018). Burden of rheumatoid arthritis in the Nordic region, 1990–2015: a comparative analysis using the Global Burden of Disease Study 2015. Scand J Rheumatol.

[2] Scott DL, Wolfe F, Huizinga TW (2010). Rheumatoid arthritis. Lancet.

[3] Agca R, Heslinga SC, Rollefstad S, Heslinga M, McInnes IB, Peters MJ (2017). EULAR recommendations for cardiovascular disease risk management in patients with rheumatoid arthritis and other forms of inflammatory joint disorders: 2015/2016 update. Ann Rheum Dis.

[4] Smolen JS, Breedveld FC, Burmester GR, Bykerk V, Dougados M, Emery P (2016). Treating rheumatoid arthritis to target: 2014 update of the recommendations of an international task force. Ann Rheum Dis.

[5] Saevarsdottir S, Wedren S, Seddighzadeh M, Bengtsson C, Wesley A, Lindblad S (2011). Patients with early rheumatoid arthritis who smoke are less likely to respond to treatment with methotrexate and tumor necrosis factor inhibitors: observations from the Epidemiological Investigation of Rheumatoid Arthritis and the Swedish Rheumatology Register cohorts. Arthritis Rheum.

[6] Ajeganova S, Andersson ML, Hafstrom I (2013). Association of obesity with worse disease severity in rheumatoid arthritis as well as with comorbidities: a long-term followup from disease onset. Arthritis Care Res (Hoboken).

[7] Bergman S, Symeonidou S, Andersson ML, Soderlin MK (2013) Alcohol consumption is associated with lower self-reported disease activity and better health-related quality of life in female rheumatoid arthritis patients in Sweden: data from BARFOT, a multicenter study on early RA. Journal. 10.1186/1471-2474-14-218 10.1186/1471-2474-14-218 PMC3734212 23879655

[8] Metsios GS, Stavropoulos-Kalinoglou A, Panoulas VF, Wilson M, Nevill AM, Koutedakis Y (2009). Association of physical inactivity with increased cardiovascular risk in patients with rheumatoid arthritis. Eur J Cardiovasc Prev Rehabil.

[9] Soderlin MK, Petersson IF, Bergman S, Svensson B (2011). Smoking at onset of rheumatoid arthritis (RA) and its effect on disease activity and functional status: experiences from BARFOT, a long-term observational study on early RA. Scand J Rheumatol.

[10] Lu B, Rho YH, Cui J, Iannaccone CK, Frits ML, Karlson EW (2014). Associations of smoking and alcohol consumption with disease activity and functional status in rheumatoid arthritis. J Rheumatol.

[11] Humphreys JH, Warner A, Costello R, Lunt M, Verstappen SMM, Dixon WG (2017). Quantifying the hepatotoxic risk of alcohol consumption in patients with rheumatoid arthritis taking methotrexate. Ann Rheum Dis.

[12] Sokka T, Hakkinen A, Kautiainen H, Maillefert JF, Toloza S, Mork Hansen T (2008). Physical inactivity in patients with rheumatoid arthritis: data from twenty-one countries in a cross-sectional, international study. Arthritis Rheum.

[13] Iversen MD, Frits M, von Heideken J, Cui J, Weinblatt M, Shadick NA (2017). Physical activity and correlates of physical activity participation over three years in adults with rheumatoid arthritis. Arthritis Care Res (Hoboken).

[14] Lindhardsen J, Ahlehoff O, Gislason GH, Madsen OR, Olesen JB, Torp-Pedersen C (2011). The risk of myocardial infarction in rheumatoid arthritis and diabetes mellitus: a Danish nationwide cohort study. Ann Rheum Dis.

[15] Petersen KE, Johnsen NF, Olsen A, Albieri V, Olsen LK, Dragsted LO (2015). The combined impact of adherence to five lifestyle factors on all-cause, cancer and cardiovascular mortality: a prospective cohort study among Danish men and women. Br J Nutr.

[16] Lacombe J, Armstrong MEG, Wright FL, Foster C (2019). The impact of physical activity and an additional behavioural risk factor on cardiovascular disease, cancer and all-cause mortality: a systematic review. BMC Public Health.

[17] Sinnathurai P, Capon A, Buchbinder R, Chand V, Henderson L, Lassere M, et al. (2018) Cardiovascular risk management in rheumatoid and psoriatic arthritis: online survey results from a national cohort study. Journal. 10.1186/s41927-018-0032-9 10.1186/s41927-018-0032-9 PMC6390588 30886975

[18] Malm K, Bremander A, Arvidsson B, Andersson ML, Bergman S, Larsson I (2016) The influence of lifestyle habits on quality of life in patients with established rheumatoid arthritis-a constant balancing between ideality and reality. Journal. 10.3402/qhw.v11.30534 10.3402/qhw.v11.30534 PMC4864849 27172513

[19] Primdahl J, Clausen J, Horslev-Petersen K (2013). Results from systematic screening for cardiovascular risk in outpatients with rheumatoid arthritis in accordance with the EULAR recommendations. Ann Rheum Dis.

[20] Arnett FC, Edworthy SM, Bloch DA, McShane DJ, Fries JF, Cooper NS (1988). The American Rheumatism Association 1987 revised criteria for the classification of rheumatoid arthritis. Arthritis Rheum.

[21] Hetland ML (2011). DANBIO–powerful research database and electronic patient record. Rheumatology (Oxford).

[22] Hafstrom I, Ajeganova S, Forslind K, Svensson B (2019) Anti-citrullinated protein antibodies are associated with osteopenia but not with pain at diagnosis of rheumatoid arthritis: data from the BARFOT cohort. Journal. 10.1186/s13075-019-1833-y 10.1186/s13075-019-1833-y PMC6360733 30717793

[23] Malm K, Bergman S, Bremander A, Larsson I, Andersson MLE (2019) Discussions of lifestyle habits as an integral part of care management: a cross-sectional cohort study in patients with established rheumatoid arthritis in Sweden. Journal. 10.1093/rap/rkz039 10.1093/rap/rkz039 PMC6827554 31701084

[24] Fleming MF, Barry KL, MacDonald R (1991). The alcohol use disorders identification test (AUDIT) in a college sample. Int J Addict.

[25] Haskell WL, Lee IM, Pate RR, Powell KE, Blair SN, Franklin BA (2007). Physical activity and public health: updated recommendation for adults from the American College of Sports Medicine and the American Heart Association. Med Sci Sports Exerc.

[26] Rausch Osthoff AK, Niedermann K, Braun J, Adams J, Brodin N, Dagfinrud H (2018). 2018 EULAR recommendations for physical activity in people with inflammatory arthritis and osteoarthritis. Ann Rheum Dis.

[27] Olsson SJ, Ekblom Ö, Andersson E, Börjesson M, Kallings LV (2016). Categorical answer modes provide superior validity to open answers when asking for level of physical activity: A cross-sectional study. Scand J Public Health.

[28] Hurst NP, Kind P, Ruta D, Hunter M, Stubbings A (1997). Measuring health-related quality of life in rheumatoid arthritis: validity, responsiveness and reliability of EuroQol (EQ-5D). Br J Rheumatol.

[29] Fries JF, Spitz P, Kraines RG, Holman HR (1980). Measurement of patient outcome in arthritis. Arthritis Rheum.

[30] Joos E, Peretz A, Beguin S, Famaey JP (1991). Reliability and reproducibility of visual analogue scale and numeric rating scale for therapeutic evaluation of pain in rheumatic patients. J Rheumatol.

[31] Strand V, Boers M, Idzerda L, Kirwan JR, Kvien TK, Tugwell PS (2011). It’s good to feel better but it’s better to feel good and even better to feel good as soon as possible for as long as possible. Response criteria and the importance of change at OMERACT 10. J Rheumatol.

[32] Duncan MJ, Kline CE, Vandelanotte C, Sargent C, Rogers NL, Di Milia L (2014). Cross-sectional associations between multiple lifestyle behaviors and health-related quality of life in the 10,000 Steps cohort. Journal.

[33] Danish Health Authority (2018) Danskernes Sundhed: Den nationale sundhedsprofil 2017 [Danes’ health: the national health profile 2017]. København

[34] The Public Health Agency of Sweden (2019) Öppna jämförelser folkhälsa 2019 [Comparisons Public Health 2019]. The Public Health Agency of Sweden (https://www.folkhalsomyndigheten.se/publicerat-material/publikationsarkiv/oe/oppna-jamforelser-folkhalsa-2019/#issuu). Accessed 26 March 2021.

[35] Perk J, De Backer G, Gohlke H, Graham I, Reiner Ž, Verschuren M (2012). European Guidelines on cardiovascular disease prevention in clinical practice (version 2012). Eur Heart J.

[36] van Wesemael TJ, Ajeganova S, Humphreys J, Terao C, Muhammad A, Symmons DPM, et al. (2016) Smoking is associated with the concurrent presence of multiple autoantibodies in rheumatoid arthritis rather than with anti-citrullinated protein antibodies per se: a multicenter cohort study. Journal. 10.1186/s13075-016-1177-9 10.1186/s13075-016-1177-9 PMC5134292 27906045

[37] Nordic Medico-Statistical Committee (2013). Health statistics for the Nordic countries 2013.

[38] The Public Health Agency of Sweden Tobaksbruk – statistik [Tobacco use - statistics]. The Public Health Agency of Sweden, (https://www.folkhalsomyndigheten.se/folkhalsorapportering-statistik/statistik-a-o/). Accessed 25 March 2021.

[39] Asmussen K-H PK-S, Petersen K-H, Schlemmer A, Hansen A, Stoltenberg M, et al. (2012) Reumatoid Artrit – Klinisk Retningslinje [Rheumatoid Arthritis - Clinical Guideline]. Dansk Reumatologisk Selskab (https://danbio-online.dk/vejledning/historik/dansk-reumatologisk-selskabs-kliniske-retningslinje/drs-kliniske-retningslinje-for-diagnostik-klassifikation-behandling-og-monitorering-af-ra). Accessed 25 Marts 2021.

[40] Malm K, Bergman S, Andersson ML, Bremander A, Larsson I (2017) Quality of life in patients with established rheumatoid arthritis: a phenomenographic study. Journal. 10.1177/2050312117713647 10.1177/2050312117713647 PMC5466281 28611920

[41] Knittle K, De Gucht V, Maes S (2012). Lifestyle- and behaviour-change interventions in musculoskeletal conditions. Best Pract Res Clin Rheumatol.

[42] Hurkmans EJ, de Gucht V, Maes S, Peeters AJ, Ronday HK, Vliet Vlieland TPM (2011). Promoting physical activity in patients with rheumatoid arthritis: rheumatologists’ and health professionals’ practice and educational needs. Clin Rheumatol.

[43] Ekdahl C, Broman G (1992). Muscle strength, endurance, and aerobic capacity in rheumatoid arthritis: a comparative study with healthy subjects. Ann Rheum Dis.

[44] Santo RCE, Fernandes KZ, Lora PS, Filippin LI, Xavier RM (2018). Prevalence of rheumatoid cachexia in rheumatoid arthritis: a systematic review and meta-analysis. J Cachexia Sarcopenia Muscle.

[45] Ollewagen T, Myburgh KH, van de Vyver M, Smith C (2021) Rheumatoid cachexia: the underappreciated role of myoblast, macrophage and fibroblast interplay in the skeletal muscle niche. Journal. 10.1186/s12929-021-00714-w 10.1186/s12929-021-00714-w PMC7931607 33658022

[46] Elkan AC, Engvall IL, Cederholm T, Hafström I (2009). Rheumatoid cachexia, central obesity and malnutrition in patients with low-active rheumatoid arthritis: feasibility of anthropometry, Mini Nutritional Assessment and body composition techniques. Eur J Nutr.

